# Primary care physicians’ familiarity, beliefs, and perceived barriers to practice guidelines in non-diabetic CKD: a survey study

**DOI:** 10.1186/1471-2369-15-64

**Published:** 2014-04-22

**Authors:** Khaled Abdel-Kader, Raquel C Greer, L Ebony Boulware, Mark L Unruh

**Affiliations:** 1 Division of Nephrology and Hypertension, Vanderbilt University, 1161 21st Ave. S., MCN S-3223, Nashville, TN 37232, USA; 2 Division of General Internal Medicine, Johns Hopkins University School of Medicine, 2024 E. Monument Street, Suite 2-600, Baltimore, MD 21287, USA; 3 Welch Center for Prevention, Epidemiology and Clinical Research, Johns Hopkins University School of Medicine, Baltimore, MD, USA; 4 Division of General Internal Medicine, Duke University School of Medicine, DUMC 104427, Durham, NC 27710, USA; 5 Division of Nephrology, University of New Mexico, DoIM MSC10-5550, Albuquerque, New Mexico 87131, USA

**Keywords:** Chronic kidney disease, Primary care physicians, Survey, Estimate glomerular filtration rate, Albuminuria, Angiotensin converting enzyme inhibitors, Angiotensin II receptor blockers, Barriers

## Abstract

**Background:**

Most non-dialysis dependent chronic kidney disease (CKD) patients are cared for by their primary care physicians (PCPs). Studies suggest many CKD patients receive suboptimal care. Recently, CKD clinical practice guidelines were updated with additional emphasis on albuminuria.

**Methods:**

We performed an internet-based, cross-sectional survey of active PCPs in the United States using the American Medical Association Physician Masterfile. We explored CKD guideline familiarity, self-reported practice behaviors, and attitudinal and external barriers to implementing guideline recommendations, including albuminuria testing.

**Results:**

Of 12,034 PCPs targeted, 848 opened a study email, 165 (19.5%) responded. Most respondents (88%) spent ≥50% of their time in clinical care. Respondents were generally in private practice (46%). Most PCPs (96%) felt that eGFR values were helpful. Approximately, 75% and 91% of PCPs reported testing for albuminuria in non-diabetic hypertensive patients with an eGFR > 60 ml/min/1.73 m^2^ and < 60 ml/min/1.73 m^2^, respectively. Barriers to albuminuria testing included a lack of effect on management, limited time, and the perceived absence of guidelines recommending testing. While PCPs expressed high levels of agreement with the definition of CKD, 30% were concerned with overdiagnosis in older adults with an eGFR in the CKD stage 3a range. Most PCPs felt that angiotensin converting enzyme inhibitor (ACEi)/ angiotensin II receptor blockers (ARBs) improved outcomes in CKD, though agreement was lower with severe vs. moderate albuminuria (78% vs. 85%, respectively, p = 0.03). Many PCPs (51%) reported being unfamiliar with CKD guidelines, but were receptive to systematic interventions to improve their CKD care.

**Conclusions:**

PCPs generally agree with CKD clinical practice guidelines regarding CKD definition and albuminuria testing. However, future interventions are necessary to improve PCPs’ familiarity with CKD guidelines, overcome barriers to albuminuria testing and, assist PCPs in targeting ACEi/ARBs to the patients most likely to benefit.

## Background

Chronic kidney disease (CKD) affects millions of Americans [1] and estimates suggest that over 50% of adults in the United States (US) will develop CKD during their lifetime [2]. Primary care providers (PCPs) deliver the majority of non-dialysis dependent CKD care and this trend is expected to continue due to limitations in the nephrologist workforce [3].

Multiple studies have documented deficiencies in PCP delivery of CKD care. These include suboptimal screening/monitoring of patients with CKD risk factors [4,5], infrequent discussions between providers and patients regarding CKD [6], suboptimal albuminuria testing in CKD patients [7,8], suboptimal blood pressure control [9], and suboptimal renin-angiotensin blockade in CKD patients with proteinuria [10,11]. In light of these deficiencies, studies have also demonstrated shortcomings in PCP knowledge of CKD risk factors [12,13] and poor awareness of Kidney Disease Outcomes Quality Initiative (KDOQI) clinical practice guidelines [14-17].

Recently, Kidney Disease Improving Global Outcomes (KDIGO) released updated CKD clinical practice guidelines [18]. For PCPs, perhaps the most salient modification is the emphasis on albuminuria as a risk stratification marker for poor outcomes. We employed the knowledge, attitudes, and behavior framework adapted by Cabana [19] to study reasons for PCP non-adherence to key components of the 2012 KDIGO CKD guidelines (i.e., assessing estimated glomerular filtration rate [eGFR] and albuminuria/proteinuria, definition of CKD, and use of angiotensin converting enzyme inhibitor [ACEi] or angiotensin II receptor blockers [ARBs] in overtly albuminuric/proteinuric patients) [18], focusing on non-diabetic CKD. We chose to concentrate on non-diabetic CKD because several studies have noted superior PCP performance in diabetic CKD (including albuminuria quantification and ACEi/ARB use) and high rates of familiarity with the American Diabetic Association’s clinical practice guidelines [7,12,20]. We also assessed interventions PCPs would find most acceptable to optimize CKD care.

## Methods

### Study design, population, and setting

We performed a web-based, cross-sectional survey of US PCPs between April and June 2013. We identified PCPs actively practicing in the US and included in the American Medical Association’s (AMA) Physician Masterfile. We emailed a simple random sample of 12,034 PCPs for this survey.

### Questionnaire design and content

The questionnaire included items assessing PCPs’ knowledge, beliefs, attitudes, self-reported behavior, and perceived barriers regarding aspects of CKD care as well as demographic and practice characteristics. Questions were developed following appraisal of the literature (KA) [6-11,13-19,21-30]. An internist and nephrologist with domain and survey expertise revised the questionnaire (RG, MU). We subsequently pilot-tested the questionnaire among 16 community- and hospital-based internists and family practitioners. We revised the questionnaire based on feedback. Several items (e.g., regarding cystatin C) were universally unfamiliar to PCPs and were removed from the survey. We then formatted the questionnaire for web-based administration and pilot tested the web-based questionnaire among 5 additional PCPs who had not reviewed previous versions.

Because studies indicate that PCPs are more likely to recognize, evaluate, and treat diabetic CKD [5,7,31], and given the added emphasis on albuminuria in the new guidelines [18], we focused questionnaire items on non-diabetic CKD and on the assessment, recognition, and treatment of albuminuria in non-diabetic CKD care. The final questionnaire featured both vignette-based and non-vignette based multiple choice questions with 33 items concerning CKD (see Additional file 1 for full questionnaire).

The initial items followed a vignette of a 73-year-old white man with well controlled hypertension treated with amlodipine and hydrochlorothiazide who presented for routine follow-up. The patient had a normal history and exam and an elevation in serum creatinine and decrement in eGFR that progressed over 12 months (1.3 mg/dl to 1.4 mg/dl and 55 ml/min/1.73 m^2^ to 50 ml/min/1.73 m^2^, respectively). We designed the vignette to simulate a common scenario that has raised concerns regarding CKD overdiagnosis due to age-related decline in kidney function [23,32-36]. Subsequent questions featured a 4-point Likert scale (ranging from strongly agree to strongly disagree, with a 5^th^ option for “Don’t Know”) to assess participants’ agreement with statements regarding the utility of eGFR, further testing with quantitative albuminuria or urinalysis, perceived limitations of urine testing due to patient burden or poor test reliability, and the presence of CKD in the described patient. Later questions more directly assessed concerns for overdiagnosis in patients with a stable eGFR between 45–59 ml/min/1.73 m^2^ and 30–44 ml/min/1.73 m^2^.

We also assessed how frequently (ranging from twice a year to never/rarely) PCPs reported testing for urine albumin/protein in non-diabetic hypertensives and potential barriers to urine testing. PCPs could select multiple barriers including patient level (e.g., cost, adherence), environmental (e.g., limited time), and attitudinal (e.g., lack of outcome expectancy) barriers.

**Figure 1:**
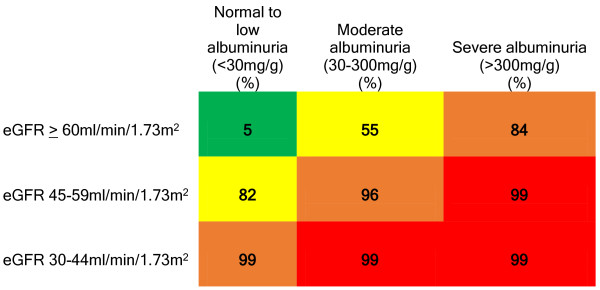
**PCPs reporting that a non-diabetic older adult with the specified characteristics has CKD.** Colors indicate CKD risk categories as classified in KDIGO guidelines. yellow – moderate, orange – high, red – very high. N = 154.

In two items, we provided PCPs with an intersecting grid of albuminuria and eGFR values to assess their beliefs regarding (a) the presence of CKD in a 65 year-old non-diabetic patient and (b) whether ACEi/ARBs would improve outcomes in a non-diabetic patient with the specified test results (e.g., Figure 1). These grids represented abbreviated versions of the 2012 KDIGO CKD classification system [18] and respondents used a drop down menu in each cell to choose their response (i.e., “yes”, “no”, or “unsure”). We also assessed patient level, environmental, and attitudinal barriers to ACEi/ARB use in non-diabetics with “macroalbuminuria”.

We assessed PCPs’ familiarity and perceived helpfulness of the KDOQI/KDIGO guidelines and their potential interest in interventions to optimize CKD care. We also collected information on PCPs’ demographics and practice characteristics.

### Survey implementation

We sent broadcast emails in 2 waves. To maximize responses [37], we sent PCPs a personally addressed introductory email that included the investigators’ university affiliations as well as a statement regarding the importance of the topic. We sent PCPs a personally addressed email invitation several days later. The invitation included a link to the web-based questionnaire and disclosed a 1-week deadline for completion. The subject heading for the broadcast emails did not mention “survey” or “questionnaire” (e.g., “Assessing the potential impact of CKD staging changes”). Emails contained statements ensuring the confidentiality of respondents and offering a $20 electronic gift card for participation. We ascertained the opened status of emails by encoding a pixel in the messages that communicated when an email was opened. We gave survey recipients 21 days to open and/or respond to the email. The University of Pittsburgh Institutional Review Board approved this study.

### Statistical analysis

We described questionnaire responses using descriptive statistics. We collapsed Likert scales into 3 categories: strongly agree/agree, strongly disagree/disagree, and don’t know. Chi-square or Fisher’s exact test was used to assess independence of categorical variables as appropriate. We employed multivariable logistic regression to quantify associations between PCP demographic/practice characteristics (independent variables) and the following dependent variables (a) reported familiarity/utility of CKD guidelines, (b) agreement with classification of stage 1–2 CKD (i.e., eGFR ≥60 ml/min/1.73 m^2^ and microalbuminuria) in a non-diabetic individual as representing CKD, and (c) concern regarding overdiagnosis in stage 3a CKD. In regression modeling, p ≤ 0.1 in univariable analysis was used to identify factors for inclusion in a multivariable backwards selection approach. Analyses were performed using Stata version 13.0 (College Station, TX) and P-values < 0.05 were considered significant.

## Results

### Baseline characteristics

Of the 12,034 PCPs, 45 had nonworking email addresses, and 11,141 did not open the study emails. Of the 848 recipients who opened a study email, 165 (19.5%) responded including 14 respondents who partially completed the survey, answering between 9 and 30 questions each. Respondent characteristics are summarized in Table 1. Nearly 90% of respondents spent at least half their time in patient care activities. The majority of respondents were internists (56%) and generally practiced in an urban (38%) or suburban (46%) locale. Nearly half of respondents (46%) were part of a private practice. Compared to targeted PCPs, respondents were more likely to be internists (47% vs 56%, respectively, p = 0.03) and had graduated medical school more recently (13% vs. 30% within 10 years, 31% vs. 28% between 11 to 20 years, 29% vs. 24% between 21 to 30 years, and 27% vs. 19% over 30 years ago, respectively, p < 0.001). Respondents were similar in gender to targeted PCPs (data not shown).

**Table 1:** Baseline respondent characteristics

	**N (%)**
Female*	45 (34.4)
**Patient care time**	
10-40%	18 (11.9)
50-70%	23 (15.2)
80-100%	110 (72.8)
**Years since medical school**	
1-10	45 (29.8)
11-20	42 (27.8)
21-30	36 (23.8)
>30	28 (18.5)
**Specialty**	
Family medicine	59 (39.1)
Internal medicine	85 (56.3)
Other (geriatrics, internal medicine-pediatrics)	7 (4.6)
**Practice location**	
Urban	57 (37.8)
Suburban	69 (45.7)
Rural	25 (16.6)
**Practice organization**	
Solo/2 person practice	19 (12.6)
Private group	51 (33.8)
Healthcare organization or HMO	28 (18.5)
University based	31 (20.5)
Government	8 (5.3)
Hospitalist	13 (8.6)
Other (corporate medical)	1 (0.7)

Continuous variables are presented as means with standard deviations.

Categorical variables are expressed as percentages and N.

N = 151 unless otherwise specified. N <165 due to 14 partial respondents who did not complete the questionnaire.

*N = 131.

### eGFR and albuminuria testing

Following the clinical vignette, 96% of PCPs agreed that an eGFR was useful in assessing kidney function. In addition, 72% and 76% of respondents agreed with the utility of a urine dipstick or a quantitative albuminuria assessment, respectively. While few respondents (5%) felt that a urine dipstick would be burdensome to the patient, 20% felt that it would not be helpful due to poor reliability. In contrast, 30% of PCPs felt a quantitative albuminuria would be burdensome to the patient and 14% felt it would not be helpful due to poor reliability.

In non-diabetic hypertensive patients with an eGFR ≥ 60 ml/min/1.73 m^2^, 75% of PCPs reported testing for albuminuria or proteinuria; 51%, 13%, and 10% of PCPs reported performing such testing annually, every 2–3 years, and twice a year, respectively. In non-diabetic hypertensive patients with an eGFR < 60 ml/min/1.73 m^2^, 91% of PCPs reported testing for albuminuria or proteinuria; 55%, 12%, and 22% of PCPs reported performing such testing annually, every 2–3 years, and twice a year, respectively. Commonly endorsed barriers to urine testing are shown in Table 2.

**Table 2:** Barriers to urine albumin/protein testing endorsed by PCPs

	**Clinical setting**	
**Barrier**	**HTN & eGFR ≥ 60 (%)**	**HTN & eGFR < 60 (%)**
No impact on management	37	24
Limited time/more urgent patient issues	25	20
Not recommended by guidelines	25	11
Cost	13	9
Poor patient adherence	5	5

Percentages do not sum to 100 as multiple responses were possible.

HTN hypertension, eGFR estimated glomerular filtration rate.

eGFR value in ml/min/1.73 m^2^.

N = 153.

### CKD definition: agreement and concerns regarding overdiagnosis

Respondents generally agreed with current guideline-based CKD definitions (Figure 1). An area where respondents expressed less agreement was in patients with an eGFR ≥ 60 ml/min/1.73 m^2^ and “microalbuminuria” (i.e., moderate albuminuria). Only 55% of PCPs felt this represented CKD.

When presented as part of the clinical vignette, 92% agreed that a chronically reduced eGFR of 50 ml/min/1.73 m^2^ represented CKD. However, when asked about overdiagnosis, 30% of PCPs agreed that classifying older adults with stable eGFRs of 45-59 ml/min/1.73 m^2^ as having CKD led to overdiagnosis, while 66% disagreed, and 4% were uncertain. In contrast, only 5% agreed that classifying older adults with eGFRs of 30–44 ml/min/1.73 m^2^ as having CKD led to overdiagnosis.

### ACEi/ARB use: outcome expectancy and barriers

The majority of respondents reported that ACEi/ARBs would improve outcomes in non-diabetics under a variety of eGFR and albuminuria categorizations (Table 3). This included 86% and 77% of PCPs when eGFR was < 60 ml/min/1.73 m^2^ and “microalbuminuria” or “macroalbuminuria” was present, respectively (microalbuminuria vs. macroalbuminuria, p = 0.03).

**Table 3:** Belief that treatment with ACEi/ARBs is beneficial in non-diabetics with the specified characteristics

	**Normal to low albuminuria (<30 mg/g) (%)**	**Moderate albuminuria (30-300 mg/g) (%)**	**Severe albuminuria (>300 mg/g) (%)**
**eGFR ≥ 60 ml/min/1.73 m**^**2**^	36*	84*	79*
**eGFR < 60 ml/min/1.73 m**^**2**^	63^†^	86^†^**	77^†^**

*eGFR ≥ 60 ml/min/1.73 m^2^: normal albuminuria vs. moderate or severe albuminuria (p < 0.001).

^†^eGFR < 60 ml/min/1.73 m^2^: normal albuminuria vs. moderate or severe albuminuria (p < 0.001).

**eGFR < 60 ml/min/1.73 m^2^: moderate albuminuria vs. severe albuminuria (p = 0.03).

eGFR estimated glomerular filtration rate.

N = 154.

Commonly endorsed barriers to ACEi/ARB use in non-diabetics with “macroalbuminuria” were adverse effects (23%), poor adherence (8%), not recommended by guidelines (8%), will not improve outcomes (7%), and more urgent patient issues/limited time (6%).

### KDOQI/KDIGO: guideline familiarity

**Figure 2:**
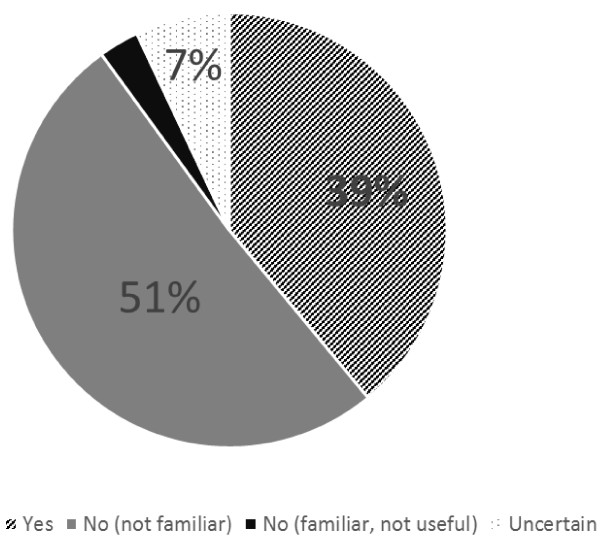
Responses to “Have the KDOQI/KDIGO CKD guidelines been helpful in managing your CKD patients?” N = 151.

Over half of respondents (Figure 2) reported the KDOQI/KDIGO guidelines were not helpful in managing their CKD patients due to a lack of familiarity.

### Interventions to enhance CKD care

PCPs were receptive to multiple systematic interventions to improve their CKD care (Table 4). Excluding continuing medical education (CME), 75% of PCPs supported an alternative intervention to improve their CKD care.

**Table 4:** Potential interventions to enhance CKD care

**Intervention to improve CKD care**	**Endorsing PCPs (%)**
Continuing medical education	74
EHR decision support	55
Academic detailing by CKD specialist	31
Collaborative practice agreements	29
Audit and feedback	28
None	3

N = 151.

Percentages do not sum to 100 as multiple responses were possible.

CKD chronic kidney disease, PCPs primary care physicians, EHR electronic health record.

### Association of PCP demographic/practice characteristics and responses

While adjusting for practice locale in logistic regression modeling, agreement with the definition of stage 1 or 2 CKD (eGFR ≥ 60 ml/min/1.73 m^2^ and moderate albuminuria) was associated with training in internal medicine (vs. family medicine, odds ratio [OR] 2.8, 95% confidence interval [CI] 1.4 - 5.9). Similarly, only training in internal medicine (vs. family medicine, OR 2.3, 95% CI 1.1 - 4.7) associated with reporting that the KDOQI/KDIGO guidelines were helpful in managing patients. None of the PCP characteristics were associated with concerns regarding overdiagnosis in stage 3a CKD.

## Discussion

We found that PCPs overwhelmingly agreed that eGFR values were helpful. Most also reported testing for albuminuria in their non-diabetic hypertensives regardless of eGFR; however, frequent barriers cited included a lack of effect on management, limited time, and the perceived absence of guidelines recommending albuminuria testing. While PCPs expressed very high agreement with the definition of CKD in patients with marked decrements in eGFR (eGFR < 45 ml/min/1.73 m^2^) or decrements in eGFR coupled with albuminuria, agreement was less robust when eGFR was > 60 ml/min/1.73 m^2^ or in CKD stage 3a without albuminuria. Similarly, a substantial minority of PCPs were concerned with overdiagnosis in older adults with an eGFR of 45-59 ml/min/1.73 m^2^. While most PCPs felt that ACEi/ARBs improved outcomes in CKD, even in the absence of albuminuria, agreement with the potential benefits of ACEi/ARBs was surprisingly highest with moderate rather than severe albuminuria. PCPs reported a lack of familiarity with KDOQI/KDIGO guidelines; however, most were receptive to systematic interventions beyond CME to improve their CKD care.

Few have previously examined PCPs’ beliefs, attitudes, and perceived barriers to eGFR reporting, albuminuria testing, CKD definitions, and ACEi/ARB use [30]. Since the publication of the KDOQI guidelines and the implementation of automated eGFR reporting, CKD has garnered increasing public and provider attention. Unfortunately, studies have documented limited improvements in CKD-related processes of care in recent years, including testing for proteinuria and use of ACEi/ARBs [7,8,38-41]. Previous surveys suggested that knowledge deficiencies [12,13,16,17,42] may contribute to continued gaps in care. In contrast to prior studies where only serum creatinine and proteinuria were used [16,42], we found that PCPs were relatively knowledgeable in identifying CKD based on eGFR and albuminuria categories. However, PCPs were less attuned to isolated albuminuria abnormalities (Figure 1). For example, while 99% of PCPs recognized that patients with a severe reduction in eGFR to 30–44 ml/min/1.73 m^2^ with “normoalbuminuria” had CKD, only 84% of PCPs recognized that patients with severe albuminuria and relatively preserved eGFR had CKD (P < 0.001).

This distinction is clinically important because the renal benefits of ACEi/ARBs in non-diabetic CKD primarily accrue in severely albuminuric patients [43,44] and the debated benefits of lower targeted blood pressure in CKD also primarily apply to albuminuric/proteinuric patients [45,46]. The under appreciation of the significance of albuminuria may be due in part to the greater emphasis on eGFR in the 2002 KDOQI guidelines. As recent studies highlight the importance of albuminuria as a marker for poor outcomes [47-49], initiatives to disseminate this information and help PCPs understand how to use albuminuria to modify their CKD care are needed. The most frequently cited barrier to testing for albuminuria was that it would have no impact on management, perhaps because responding PCPs presumed such patients would already be receiving ACEi/ARBs. However, recent data suggests that active ACEi/ARB use is unlikely to fully explain the low rates of albuminuria/proteinuria testing [7,38,50]. We suspect limited time and a lack of clarity on how to integrate information regarding albuminuria in developing non-diabetic CKD care goals is also contributing to low rates of testing.

Prior studies have documented suboptimal ACEi/ARB use in CKD patients with proteinuria [10,11]. We found that most PCPs felt that non-diabetic patients with an eGFR < 60 ml/min/1.73 m^2^ would benefit from an ACEi/ARB regardless of proteinuria. These patterns may be due in part to greater familiarity with recommendations from alternative guidelines [51] that favor ACEi/ARB use as first line treatment in patients with CKD and hypertension regardless of albuminuria. Of note, similar to prior studies [15-17], we confirmed low familiarity with KDOQI/KDIGO CKD guidelines.

Our findings support efforts to disseminate the KDIGO CKD guidelines and to provide PCPs with guidance regarding the use of albuminuria and other factors to identify patients at high risk for poor outcomes [49] and most likely to benefit from ACEi/ARBs. The most commonly endorsed barrier to ACEi/ARB use was adverse effects. Studies are needed to determine whether targeting high-risk CKD patients who may be more likely to achieve benefits from these medications [52] could reduce adverse effects and improve outcomes in older adults with multiple medications (e.g., NSAIDs, diuretics) [53] compared to other less nuanced recommendations [51,54]. Such an approach could also help PCPs identify a smaller cohort of ACEi/ARB intolerant patients with a higher likelihood to benefit from the use of these medications. In the setting of severe albuminuria, these individuals may benefit from nephrologist input to determine if alternative strategies can allow for ACEi/ARB use.

Although a previous survey of PCPs noted patient non-adherence and the cost of medications as major obstacles to appropriate care and improving patient outcomes [16], few respondents in our survey identified these as obstacles that prevent them from ordering urine albumin tests or ACEi/ARBs. This may reflect differences in the underlying populations the responding PCPs serve and changes in the cost of medications, including the greater availability of generic ACEi/ARBs.

Many of the barriers we identified are potentially addressable using systematic interventions. For example, decision support within an electronic health record could alert a provider as s/he orders a serum creatinine that a patient with possible CKD does not have a documented urine albumin test and is not receiving an ACEi/ARB. The alert could also provide references to guidelines and the studies that informed those guidelines. Similarly, collaborative practice agreements or audit and feedback could target high-risk CKD patients to ensure they receive optimal, evidence-based care. Combining several of these interventions while targeting patients who are most likely to benefit from ACEi/ARBs [52] may prove even more impactful. Most PCPs were receptive to at least one of these approaches and studies are needed to understand whether such strategies can improve care without substantially disrupting PCP workflow [55].

Our findings should be interpreted in light of several limitations. First, although we employed best practices for electronic surveys [37], the survey response rate was low, which may limit generalizability. Responders may have substantively differed from non-responders. For example, they may have had an interest in kidney disease and been more knowledgeable than the average PCP. Respondents were more likely to be internists and had graduated medical school more recently, characteristics that may suggest greater familiarity with CKD guidelines [13,16,42,56]. Indeed, self-reported guideline familiarity was modestly higher than in prior PCP survey studies [12,15,16], although it remained less than 50%. Second, the majority of targeted PCPs never opened a study email. While we were able to exclude individuals with non-valid emails, we could not determine whether an unopened message was due to an unmonitored email account, automated filtering into an infrequently checked folder (e.g., junk), a choice not to open the email based on the subject matter or sender, or other reasons. Including individuals with unopened emails would lower the response rate. Third, PCPs may not have accurately recalled their practice patterns or may have attempted to provide what they perceived as the correct answer. To mitigate this risk, we framed survey questions as assessing beliefs rather than knowledge. Finally, there were additional questions that would have been helpful to examine such as the perceived utility of cystatin C [18]. Nevertheless, our findings provide novel information regarding PCPs’ beliefs and reported barriers to adoption of recent CKD practice guidelines. Future work to examine how local resources, healthcare environments, and patient characteristics influence providers’ beliefs, perceived barriers, and practice patterns is warranted.

## Conclusions

Our survey found that PCPs value eGFR measures and are able to recognize CKD based on a decrement in eGFR. However, they were less familiar with albuminuria abnormalities and endorsed several barriers to testing for albuminuria in non-diabetics including lack of outcome expectancy, lack of guideline familiarity, and lack of time. While PCPs believed that ACEi/ARBs improve outcomes in various eGFR/albuminuria stages of CKD, future interventions will be needed to help operationalize how to identify patients at high risk for progression and who are more likely to benefit from ACEi/ARBs. Fortunately, most PCPs appear receptive to interventions to improve their CKD care.

## Abbreviations

ACEi: Angiotensin converting enzyme inhibitor; AMA: American Medical Association; ARB: Angiotensin receptor blocker; CME: Continuing medical education; CI: Confidence interval; CKD: Chronic kidney disease; eGFR: Estimated glomerular filtration rate; KDIGO: Kidney Disease Improving Global Outcomes; KDOQI: Kidney Disease Outcomes Quality Initiative; PCP: Primary care physician; US: United States.

## Competing interests

The authors declare they have no competing interests.

## Authors’ contributions

KA, RG, MU conceptualized the study. KA obtained funding. KA, RG, MU acquired the data. KA analyzed the data. KA, RG, LEB, MU contributed to interpretation and manuscript preparation. All authors read and approved the final manuscript.

## Pre-publication history

The pre-publication history for this paper can be accessed here:

http://www.biomedcentral.com/1471-2369/15/64/prepub

## Supplementary Material

Additional file 1 PCP Questionnaire. Click here for file
