# Supplementary material for: Primary care physicians’ familiarity, beliefs, and perceived barriers to practice guidelines in non-diabetic CKD: a survey study
Source: BMC Nephrol. 2014 Apr 22;15:64. doi: 10.1186/1471-2369-15-64 (PMC4021215; doi:10.1186/1471-2369-15-64)
Supplement: Additional file 1 — PCP Questionnaire. [file 1471-2369-15-64-S1.doc]

**Redefining Chronic Kidney Disease: a Survey of PCPs**

There has been a recent change in the classification/staging of chronic kidney disease (CKD). To better understand the potential impact of the proposed shift on CKD care, researchers at the University of Pittsburgh and the University of New Mexico are requesting your assistance in completing this brief research survey.

The survey can be completed from memory and should take approximately 5-7minutes. Participants, who complete the survey, will have the option of entering their email address at the end of the survey to receive a $20 Amazon e-gift card within 4-6 weeks. Your email address will not be used for any purpose other than providing the e-gift card.

This survey is anonymous and is being distributed to PCPs identified through the AMA physician masterfile. Aggregate information from the survey will be released but your individual responses will not be identifiable or linked to your email address. There are no foreseeable risks or direct benefits associated with this research. Completion of this survey is completely voluntary and you may withdraw at any time.

Please complete the survey in 1 sitting by May 24, 2013. Please scroll to the bottom of each page to answer all questions.

**Thank you for your participation!**

A 73 year-old white man with hypertension and dyslipidemia presents to your office for routine follow-up. He checks his blood pressure at home on a daily basis and reports it consistently measures in the 130-140/80-90 mmHg range over the past several months. He is otherwise without complaints.

He currently takes amlodipine 5mg daily, hydrochlorothiazide 25mg daily, atorvastatin 20mg daily, and is tolerating them well. He reports excellent medication adherence and denies any herbal or over the counter medications including NSAIDs. He has no allergies or adverse effects related to medications. He does not smoke or drink and is a retired engineer.

His physical exam today reveals: temperature 37 C, pulse 82/minute, blood pressure 136/86 mmHg, respirations 10/minute, weight 160 lb, and height 5 feet 10 inches. The remainder of his exam is benign.

Labs: serum creatinine 1.4 mg/dl, eGFR 50 ml/min/1.73m^2^ (creatinine a year ago: 1.3 mg/dl, eGFR 55 ml/min/1.73m^2^), BUN of 14 mg/dl. Serum chemistries and CBC were otherwise normal.

**1. Based on the above case, please indicate your level of agreement/disagreement with the following statements.**

|  | Strongly Agree | Agree | Disagree | Strongly  Disagree | Don’t  Know |
| --- | --- | --- | --- | --- | --- |
| Measurement of the patient’s serum creatinine was useful in assessing his kidney function. |  |  |  |  |  |
| Reporting an estimated glomerular filtration rate (eGFR) was useful in assessing his kidney function. |  |  |  |  |  |
| Ordering a quantitative albuminuria measurement would be useful. |  |  |  |  |  |
| Ordering a quantitative albuminuria measurement would be burdensome. |  |  |  |  |  |
| Ordering a quantitative albuminuria measurement would not be helpful due to poor reliability. |  |  |  |  |  |
| Ordering a urinalysis would be useful. |  |  |  |  |  |
| Ordering a urinalysis would be burdensome to the patient. |  |  |  |  |  |
| Ordering a urinalysis would not be helpful due to poor reliability. |  |  |  |  |  |
| The patient has chronic kidney disease (CKD). |  |  |  |  |  |

N=165

**2.** The following question asks about your beliefs. Please use the drop down boxes to select your response for each eGFR + albuminuria combination.

|  | Normoalbuminuria  (<30mg/g) | Microalbumbinuria  (30-300mg/g) | Macroalbuminuria  (>300mg/g) |
| --- | --- | --- | --- |
| a **chronic eGFR > 60** ml/min/1.73m^2^ | Yes (ACEi/ARBs improve outcomes)  No  (ACEi/ARBs don’t improve outcomes)  Unsure | Yes  No  Unsure | Yes  No  Unsure |
| a **chronic eGFR < 60** ml/min/1.73m^2^ | Yes  No  Unsure | Yes  No  Unsure | Yes  No  Unsure |

I believe that treatment with ACE inhibitors or ARBs improve outcomes in **non-diabetic** patients with…

N=154

**3.** The following question asks about your beliefs.

Please indicate whether you believe a non-diabetic older adult (age > 65 years) with the following test results has CKD...

|  | Normoalbuminuria  (<30mg/g) | Microalbumbinuria  (30-300mg/g) | Macroalbuminuria  (>300mg/g) |
| --- | --- | --- | --- |
| a **stable eGFR > 60** ml/min/1.73m^2^ | Yes  (CKD present)  No  Unsure | Yes  No  Unsure | Yes  No  Unsure |
| a stable **eGFR between 45-59** ml/min/1.73m^2^ | Yes: 127  No: 19  Unsure: 8 | Yes: 148  No: 2  Unsure: 4 | Yes  No  Unsure |
| a stable **eGFR between 30-44** ml/min/1.73m^2^ | Yes  No  Unsure | Yes  No  Unsure | Yes  No  Unsure |

N=154

4. The following question asks about your beliefs. Please indicate the extent to which you agree/disagree with the statements.

I believe that in non-diabetic older adults (age >65 years) …

|  | Strongly Agree | Agree | Disagree | Strongly  Disagree | Don’t  Know |
| --- | --- | --- | --- | --- | --- |
| classifying patients with a stable eGFR between 45-59  ml/min/1.73m^2^ as having CKD leads to overdiagnosis |  |  |  |  |  |
| classifying patients with a stable eGFR between 30-44  ml/min/1.73m^2^ as having CKD leads to overdiagnosis. |  |  |  |  |  |

N=153

**Please select one answer.**

**5**. In your **non-diabetic** patients with an **eGFR > 60** ml/min/1.73m^2^ and **hypertension**, how frequently do you measure urine albumin or urine protein (e.g., a urine albumin-to-creatinine ratio, urine protein-to-creatinine ratio, or urine dipstick for protein)?

□ Twice a year

□ Once a year

□ Once every 2-3 years

□ Never/Rarely

□ Other (please specify)

N=153

**6**. In **non-diabetic** patients with an **eGFR > 60** ml/min/1.73m^2^ and **hypertension**, please indicate reasons why you sometimes don’t test for urine albumin or protein.

Please select all that apply.

□ Cost to the patient

□ Do not believe it will affect management

□ Poor patient adherence

□ Not recommended by existing guidelines

□ Limited time/more urgent patient issues

□ None of the above, I always check a urine albumin/protein

□ Other (please specify)

N=153

**7**. In your **non-diabetic** patients with an **eGFR < 60** ml/min/1.73m^2^ and **hypertension**, how frequently do you measure urine albumin or urine protein (e.g., a urine albumin-to-creatinine ratio, urine protein-to-creatinine ratio, or urine dipstick for protein)?

□ Twice a year

□ Once a year

□ Once every 2-3 years

□ Never/Rarely

□ Other (please specify)

N=153

**8**. In **non-diabetic** patients with an **eGFR < 60** ml/min/1.73m^2^ and **hypertension**, please indicate reasons why you sometimes don’t test for urine albumin or protein.

Please select all that apply.

□ Cost to the patient

□ Do not believe it will affect management

□ Poor patient adherence

□ Not recommended by existing guidelines

□ Limited time/more urgent patient issues

□ None of the above, I always check a urine albumin/protein

□ Other (please specify)

N=153

**9.** In **non-diabetic** patients with documented **macroalbuminuria**, please indicate reasons you sometimes don’t prescribe an ACE inhibitor or ARB.

Please select all that apply.

□ Cost to the patient

□ Do not believe it will improve outcomes

□ Poor patient adherence

□ Potential adverse effects

□ Not recommended by existing guidelines

□ Limited time/more urgent patient issues

□ Communications barriers with the patient

□ None of the above, I always use an ACEi or ARB

□ Other (please list)

N=151

**10**. Which of the following interventions would most appeal to you to help optimize your care of patients with CKD?

Please select all that apply.

□ Continuing medical education lectures (CME)

□ Collaborative practice agreements (i.e., practice based management with the help of pharmacists or

physician extenders)

□ Electronic health record clinical decision support (e.g., real-time clinical reminders within workflow): 83

□ Academic detailing by CKD specialist

□ Audit and feedback on clinical performance

□ None of the above

□ Other (Please specify:)

N=151

**11.** Have the KDOQI/KDIGO CKD guidelines been helpful in managing your CKD patients?

□Yes

□ No, I’m not familiar with them

□ No. I’m familiar with them, but they are not useful in my practice

□ Unsure

N=151

**Please see manuscript table 1 for PCP demographic survey responses**

**12**. Which best describes your field of training? (select one)

□ Family Medicine

□ Internal Medicine

□ Internal Medicine/Pediatrics

□ Geriatrics

□ Other (please describe):

**13**. Is your practice setting primarily: (select one)

□ Urban

□ Suburban

□ Rural

**14**. Which best describes your primary practice setting? (select one)

□ Solo/2 person

□ Private group

□ Healthcare organization or HMO

□ University based

□ Government

□ Hospitalist

□ Other (please specify):

**15**. Approximately what percent of your time is spent in direct patient care? ____%

**16**. How many years has it been since you graduated medical school? ____ yr(s)

**17.** Please select your gender.

□ Female

□ Male

**Thank you for your participation!**

Please enter your email address below to receive a $20 Amazon e-gift card. Please allow 4-6 weeks for delivery.
